# The Best Catheter for Patients with Enlarged Prostate

**Published:** 1873-03-01

**Authors:** Edmund Andrews

**Affiliations:** Professor of Surgery in Chicago Medical College


### THE BEST CATHETER FOR PATIENTS WITH ENLARGED PROSTATE.

BY EDMUND ANDREWS, M. D., PROFESSOR OF SURGERY IN CHICAGO MEDICAL COLLEGE.

It seems not to be generally known, while men are inventing costly and complicated silver catheters for cases of enlarged prostate, that there is already in existence a very simple and inexpensive one, which is as near to absolute perfection as human inventions usually get. I refer to Mercier's *sonde coudee.*

Apparently this instrument is as unknown in England as in the United States; for, while some of us have used it in Chicago for the last six years, the London *Lancet* of November gives an engraving of it, as one of the valuable things with which English surgeons are yet unacquainted.

It is very surprising that an article so valuable is so little known. In obtaining my last supply I ransacked New York and Philadelphia in vain, and was forced to send to London. Even there, by purchasing two dozen, I exhausted the stock of that great city. Paris probably might have furnished them, only it was inaccessible in consequence of the late siege.

For cases of enlarged prostate, Mercier's *sonde coudee* is a splendid instrument. It slips into the bladder without resistance or pain, when every effort with the ordinary silver instrument only brings blood. The patient himself can use it with ease, safety and comfort, and thus prevent much of the distress and misery which old men often suffer from retention of urine. It is not alone in cases of enlarged prostate that this instrument is useful. It is superior to any other for nineteen-twentieths of the male patients by whom a catheter is required, and will, when it is better known, almost totally supplant the other forms now in use.

The *sonde coudee* is very simple. It consists of a black, flexible catheter, having two peculiarities:

1st. Its walls are about twice or thrice thicker than the ordinary catheter, so that it possesses a mixed flexibility and firmness, which enable it to conform easily to the curves of the urethra, and yet it requires no wire when inserted.

2d. Half an inch from the tip it is bent forward at an obtuse angle. This curve is the peculiarity which enables it to slip past the overhanging central lobe of the prostate. Its extremity strikes the lobe obliquely, lifting it a little, while the flexibility of the instrument allows it to bend forward and evade the obstacle with the greatest ease.

These catheters cost a dollar each at retail. When the demand shall cause them to be manufactured more abundantly, they will probably be retailed at fifty cents. At present they are to be found in this city only at E. H. Sargent's; but Degenhardt, and Bliss & Torrey, have ordered them from Europe, and will soon have a full supply. If a brisk demand springs up on the part of the profession, they will soon be kept in every drug store.

There is a very ingenious silver prostatic catheter, which has its curved portion made of movable joints. This does excellently well, but is necessarily somewhat expensive; and one cannot help feeling that if the instrument becomes weakened by age and use, or is faultily constructed in the first place, the terminal joints may some time drop off in the bladder. However, this could probably be guarded against by always trying its strength with the hand before introducing it.

There is sometimes sold a prostatic catheter of metal, whose peculiarity consists in a short curve at the end. This is better for such purposes than the old-fashioned silver instrument, but by no means equal to the two kinds before mentioned.

No. 6 Sixteenth street, Chicago.

---

A Substitute for Quinia.—Carbazotate of ammonia is given, with successful results, in France, in place of quinine. It is given in 2 *ctgr.* doses, in the form of a pill.

